# Supplementary material for: Bactericidal effect of tetracycline in E. coli strain ED1a may be associated with ribosome dysfunction
Source: Nat Commun. 2024 Jun 5;15:4783. doi: 10.1038/s41467-024-49084-5 (PMC11153495; doi:10.1038/s41467-024-49084-5)
Supplement: Supplementary file 1 — Supplementary Information [file 41467_2024_49084_MOESM1_ESM.pdf]

Supplementary Information

for

Bactericidal effect of tetracycline in *E. coli* strain ED1a may be associated with ribosome dysfunction

Iskander Khusainov, Natalie Romanov, Camille Goemans, Beata Turoňová, Christian E. Zimmerli, Sonja Welsch, Julian D. Langer, Athanasios Typas, Martin Beck

This file contains:

2 Supplementary Tables

6 Supplementary Figures

Supplementary References

Supplementary Tables

Supplementary Table 1. Single particle cryo-EM data collection and data processing statistics.

	E. coli ED1a 70S·TET 30S head focused	*E. coli* ED1a 70S·TET 30S body focused	*E. coli* ED1a 70S·TET 50S focused	*E. coli* K-12 70S·mRNA·A, P,E	*E. coli* K-12 70S·mRNA·A, P,E
Magnification	105,000 X	105,000 X	105,000 X	105,000 X	105,000 X
Voltage (kV)	300	300	300	300	300
Exposure dose ($e^{-}/Å^{2}$)	40	40	40	40	40
Number of frames	40	40	40	40	40
Defocus range (μm)	-0.25 to -2.5	-0.25 to -2.5	-0.25 to -2.5	-0.25 to -2.5	-0.25 to -2.5
Pixel size (Å/px)	0.837	0.837	0.837	0.837	0.837
No. of micrographs	10,568	10,568	10,568	12,022	16,559
No. of extracted particles	1,211,457	1,211,457	1,211,457	1,167,533	1,020,111
Final no. of particles	835,256	835,256	835,256	80,169	132,290
FSC threshold	0.143	0.143	0.143	0.143	0.143
Map resolution (Å)	2.83	2.83	2.75	3.0	3.0
Accession numbers	EMDB-19206	EMDB-19207	EMDB-19208	EMDB-18041	EMDB-18042

Supplementary Table 2. Cryo-ET data collection and sub-tomogram averaging statistics.

**Parameter**	*E. coli* combined	*E. coli* K-12 30 min LB	*E. coli* K-12 30 min TET	*E. coli* ED1a 30 min LB	*E. coli* ED1a 30 min TET
**Data collection and processing**					
Magnification	53,000 X	53,000 X	53,000 X	53,000 X	53,000 X
Voltage (kV)	300	300	300	300	300
Exposure dose ($e^{-}/Å^{2}$)	120-130	120-130	120-130	120-130	120-130
Defocus range (μm)	-1.5 to -4.5	-1.5 to -4.5	-1.5 to -3.5	-1.5- to 4.0	-1.5 to -3.5
Pixel size (Å/px)	1.697	1.697	1.697	1.697	1.697
No. of collected tilt series	264	87	82	51	44
No. of used tomograms	104	34	17	26	27
No. of extracted particles	208,000	68,000	34,000	52,000	54,000
Final no. of particles	75,670	33,250	15,744	30,548	21,818
FSC threshold	0.143	0.143	0.143	0.143	0.143
Map resolution (Å)	5.3	8.5	8.5	7.5	7.0
Accession numbers	EMDB-18036	EMDB-18037 EMPIAR-11945	EMDB-18038 EMPIAR-11946	EMDB-18039 EMPIAR-11947	EMDB-18040 EMPIAR-11948

Supplementary Figures

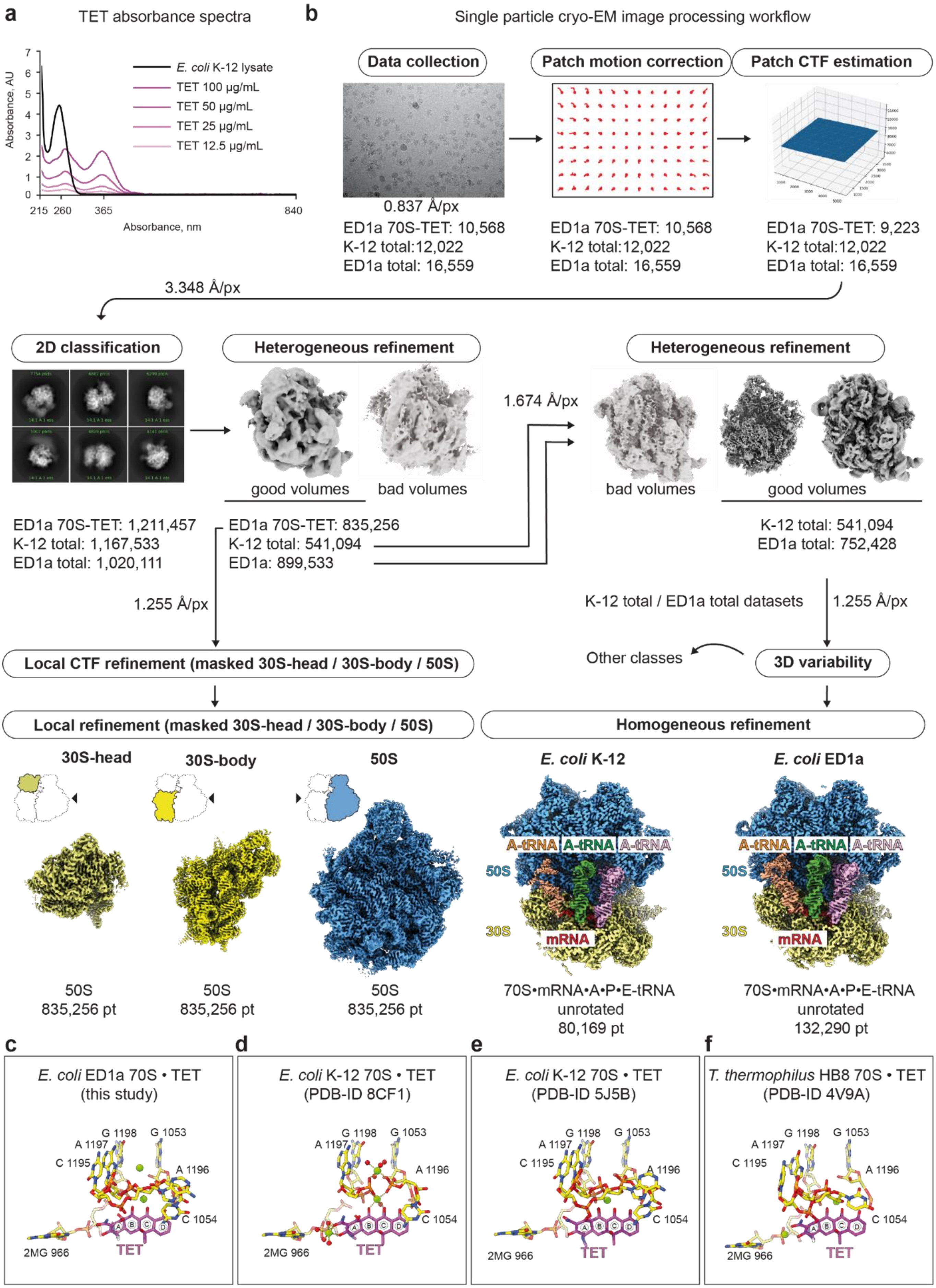
a
TET absorbance spectra
E. coli K-12 lysate
TET 100 μg/mL
TET 50 μg/mL
TET 25 μg/mL
TET 12.5 μg/mL
Absorbance, AU
215 260 365 840
Absorbance, nm
b
Single particle cryo-EM image processing workflow
Data collection
Patch motion correction
Patch CTF estimation
0.837 Å/px
ED1a 70S-TET: 10,568
K-12 total:12,022
ED1a total: 16,559
ED1a 70S-TET: 10,568
K-12 total:12,022
ED1a total: 16,559
ED1a 70S-TET: 9,223
K-12 total:12,022
ED1a total: 16,559
3.348 Å/px
2D classification
Heterogeneous refinement
good volumes
bad volumes
ED1a 70S-TET: 1,211,457
K-12 total: 1,167,533
ED1a total: 1,020,111
ED1a 70S-TET: 835,256
K-12 total: 541,094
ED1a: 899,533
1.674 Å/px
Heterogeneous refinement
bad volumes
good volumes
K-12 total: 541,094
ED1a total: 752,428
1.255 Å/px
K-12 total / ED1a total datasets
1.255 Å/px
Local CTF refinement (masked 30S-head / 30S-body / 50S)
Other classes
3D variability
Local refinement (masked 30S-head / 30S-body / 50S)
Homogeneous refinement
30S-head
30S-body
50S
E. coli K-12
E. coli ED1a
A-tRNA A-tRNA A-tRNA
50S
30S
mRNA
50S
835,256 pt
50S
835,256 pt
50S
835,256 pt
70S•mRNA•A•P•E-tRNA
unrotated
80,169 pt
70S•mRNA•A•P•E-tRNA
unrotated
132,290 pt
c
E. coli ED1a 70S • TET
(this study)
d
E. coli K-12 70S • TET
(PDB-ID 8CF1)
e
E. coli K-12 70S • TET
(PDB-ID 5J5B)
f
T. thermophilus HB8 70S • TET
(PDB-ID 4V9A)
G 1198
G 1053
A 1197
C 1195
A 1196
C 1054
2MG 966
TET

Supplementary Figure 1. Structural analysis of the ribosomes reveals identical structure of TET-binding pocket in bacteria.

a The absorbance spectrum of the cell lysate (black) and TET, titrated in phosphate buffered saline to concentrations ranging from 12.5 to 100 μg/ml (shades of purple). TET has an absorption peak at 365 nm, which is not present in the untreated cell lysate. Source data are provided as a Source Data file.

b Classification strategy used to obtain 70S structures using single particle cryo-EM. For simplicity, the intermediate reconstructions (gray) are shown only for *E. coli* K-12 dataset. The numbers denoted below the images correspond to the micrographs (mic) and particles (pt) analyzed at each step for ED1a 70S·TET, K-12 70S·mRNA·A,P,E, and ED1a 70S·mRNA·A,P,E datasets. The final reconstructions are shown for each dataset.

c – f Comparison of the TET binding sites of *E. coli* ED1a 70S·TET structure from the current study (**c**) with published structures of the 70S·TET complex from *E. coli* K-12 (**d,e**) [1,2] and *T. thermophilus* (**f**) [3].

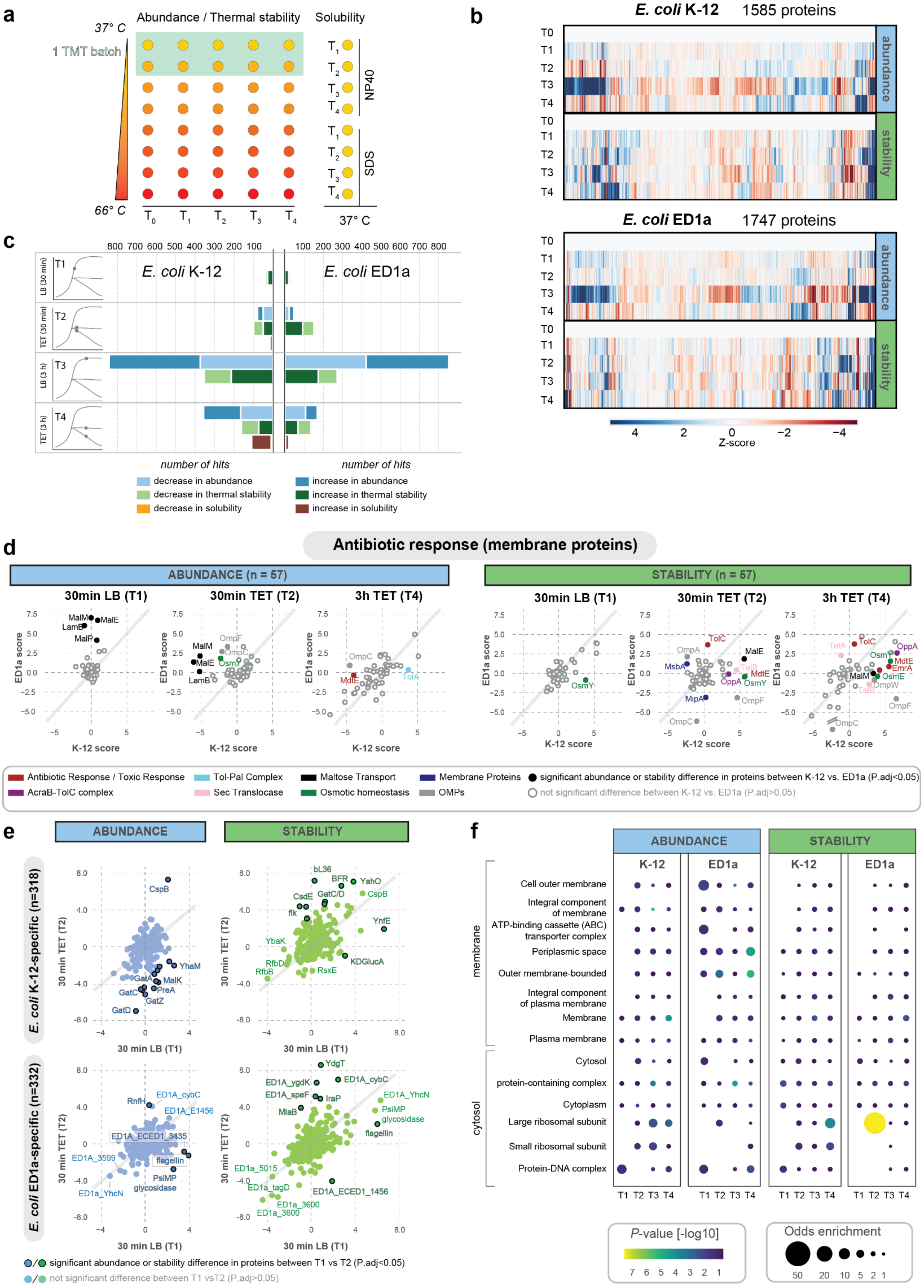

a
Abundance / Thermal stability
Solubility
37° C
1 TMT batch
66° C
NP40
SDS
37° C
b
E. coli K-12 1585 proteins
abundance
stability
E. coli ED1a 1747 proteins
Z-score
c
E. coli K-12
E. coli ED1a
LB (30 min)
TET (30 min)
LB (3 h)
TET (3 h)
number of hits
decrease in abundance
decrease in thermal stability
decrease in solubility
increase in abundance
increase in thermal stability
increase in solubility
d
Antibiotic response (membrane proteins)
ABUNDANCE (n = 57)
STABILITY (n = 57)
30min LB (T1)
30min TET (T2)
3h TET (T4)
ED1a score
K-12 score
Antibiotic Response / Toxic Response
Tol-Pal Complex
Maltose Transport
Membrane Proteins
significant abundance or stability difference in proteins between K-12 vs. ED1a (P.adj<0.05)
AcraB-TolC complex
Sec Translocase
Osmotic homeostasis
OMPs
not significant difference between K-12 vs. ED1a (P.adj>0.05)
e
ABUNDANCE
STABILITY
E. coli K-12-specific (n=318)
E. coli ED1a-specific (n=332)
30 min TET (T2)
30 min LB (T1)
significant abundance or stability difference in proteins between T1 vs T2 (P.adj<0.05)
not significant difference between T1 vsT2 (P.adj>0.05)
f
ABUNDANCE
STABILITY
K-12
ED1a
membrane
cytosol
Cell outer membrane
Integral component of membrane
ATP-binding cassette (ABC) transporter complex
Periplasmic space
Outer membrane-bounded
Integral component of plasma membrane
Membrane
Plasma membrane
Cytosol
protein-containing complex
Cytoplasm
Large ribosomal subunit
Small ribosomal subunit
Protein-DNA complex
P-value [-log10]
Odds enrichment

Supplementary Figure 2 2D-TPP identifies changes in abundance and thermal stability for proteins involved in antibiotic response and several uncharacterized proteins.

a Schematic setup of 2D-TPP analysis applied in this study. For abundance and thermal stability measurements (left), protein fractions were used upon heating from 37 °C to 66 °C. For solubility measurements, the experiments were done in the presence of NP40 or SDS, respectively, at a constant temperature of 37 °C. Each sample was prepared in four biological replicates.

b Heatmap representation of abundance (upper block), and thermal stability (lower block) changes of proteins identified in the TPP screen. Coloring corresponds to the calculated z-score ranging from -4 (red) to +4 (blue). The timepoint T3 (3 h LB) shows drastic proteome rearrangements, likely due to a shift to stationary phase of both strains.

c Bar plot illustrating the number of significantly affected proteins in abundance (blue shades), thermal stability (green shades), and solubility (orange/brown shades) for *E. coli* K-12 (left) and *E. coli* ED1a (right) for all analyzed timepoints. Timepoints T1 and T3 represent untreated cells grown for 30 min and 3 h, respectively. Timepoints T2 and T4 represent TET treated cells grown for 30 min and 3 h, respectively. The scores are calculated relative to the control condition T0 (untreated cells, 0 min LB).

d Scatter plot of abundance (left) and stability values (right) for annotated membrane proteins involved in antibiotic response according to gene ontology mapping for T1 (30 min LB), T2 (30 min TET) and T4 (3 h TET), respectively, comparing K-12 scores (*x*-axis) against ED1a scores (*y*-axis). A two-sided *t*-test was applied based on the effect size distribution resulting from the respective K-12 vs. Ed1a scores. *P*-values were adjusted using the Benjamini-Hochberg method. Each dot represents a protein; if it is highlighted in color, it signifies that there is a significant difference between K-12 and ED1a scores (P_{adj}<0.05, confidence interval 0.95). Exact p-values for each protein can be found in Supplementary Data 1. The color scheme is explained in the legend below.

e Scatter plot of abundance (left) and stability values (right) for protein hits detected only in K-12 (upper panels) or ED1a (lower panels) samples, comparing T1 (30 min LB) scores (*x*-axis) against T2 (30 min TET) scores (*y*-axis). A two-sided *t*-test was applied based on the effect size distribution resulting from the respective K-12 vs. Ed1a scores. *P*-values were adjusted using the Benjamini-Hochberg method. Each dot represents a protein; highlighted black contours indicate a significant difference between T1 and T2 scores (P_{adj}<0.05, confidence interval 0.95). Exact p-values for each protein can be found in Supplementary Data 1. The color scheme is explained in the legend below the figure panel.

f Gene ontology (GO) enrichment plot for cellular compartments, with compartments ordered according to their physical location on the outer membrane – cytosol axis for all analyzed timepoints. The enrichment for each GO-term in the significant protein set (global & local FDR < 0.05) in each respective condition (*x*-axis) is calculated using the Fisher Exact Test (two-sided), relative to the insignificant portion of proteins in each condition. Bubbles are displayed for GO-terms that are enriched in at least one condition (*P*<0.01) and have at least 10 protein components significantly affected in the dataset. The bubble size indicates the odds enrichment, whereas the bubble color reflects the *P*-value (-log10 scale).

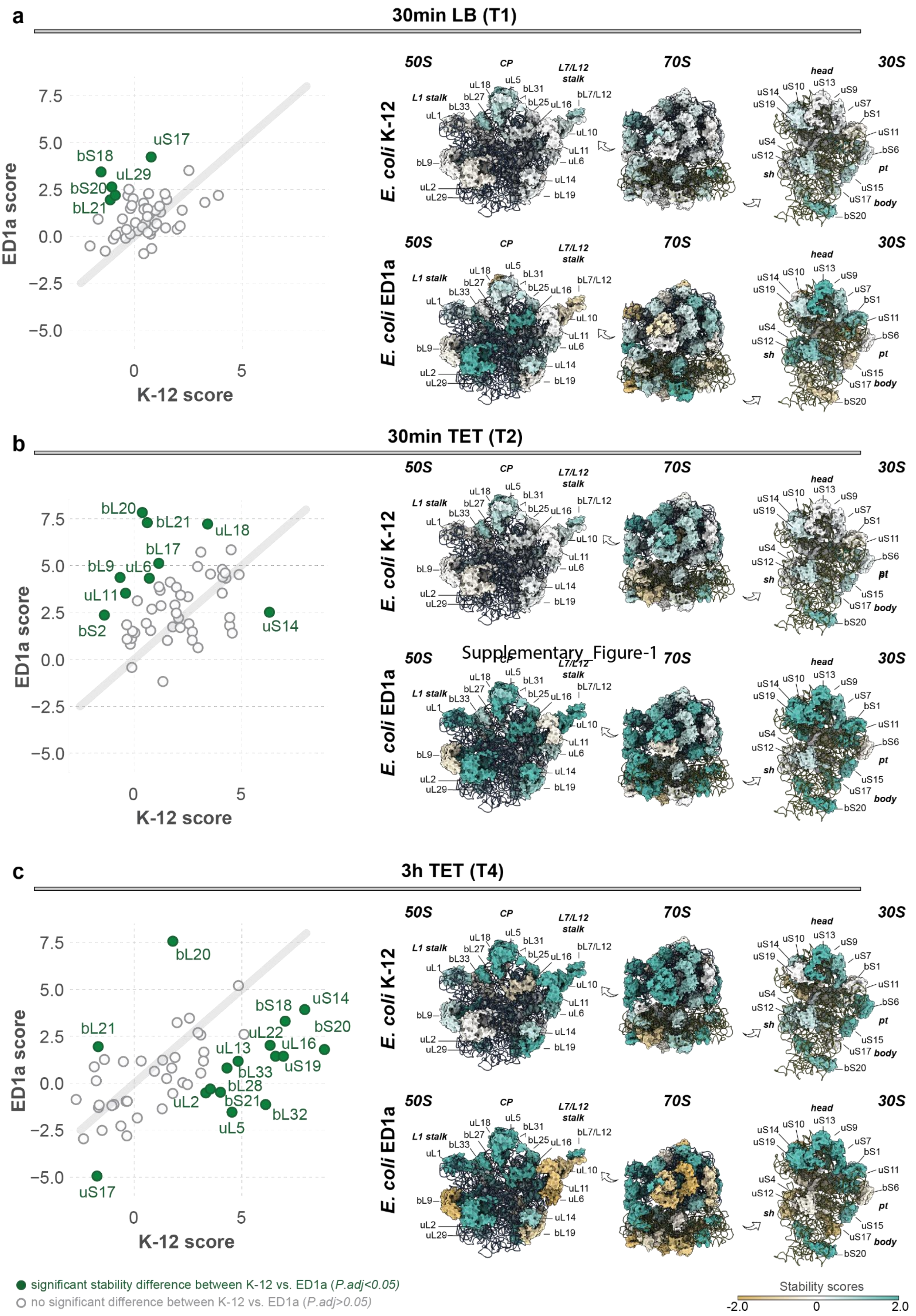
a
30min LB (T1)
b
30min TET (T2)
c
3h TET (T4)
ED1a score
K-12 score
50S
70S
30S
CP
L7/L12 stalk
L1 stalk
head
sh
pt
body
E. coli K-12
E. coli ED1a
Supplementary_Figure-1
bS18
uS17
uL29
bS20
bL21
bL20
uL18
bL17
bL9
uL6
uL11
bS2
uS14
bS18
bS20
uL22
uL13
uL16
bL33
uS19
bL28
bS21
uL2
bL32
uL5
significant stability difference between K-12 vs. ED1a (P.adj<0.05)
no significant difference between K-12 vs. ED1a (P.adj>0.05)
Stability scores
-2.0
0
2.0

Supplementary Figure 3. TPP values mapped on ribosome structures show a thermal stability increase for interface r-proteins and a decrease for peripheral r-proteins in *E. coli* ED1a upon TET treatment.

a – **c** Left-hand side: scatter plot of stability values for r-proteins for (A) T1 (30 min LB), (B) T2 (30 min TET), (C) for T4 (3 h TET) comparing K-12 *Z*-scores (*x*-axis) against ED1a scores (*y*-axis). Each dot represents a protein; significant differences between K-12 and ED1a scores are colored in green ($P_{adj}<0.05$). Right-hand side: structure of the 50S, 70S, and 30S ribosome structures in K-12 (upper) and ED1a (lower), shaded according to the respective thermal stability score values in *E. coli* K-12 and ED1a, respectively. The PDB model was combined from coordinates fetched from PDB 3J7Z, PDB 7K00, and PDB 6H4N. Proteins with increased stability are colored turquoise, proteins with decreased stability are colored tan, and proteins with unchanged stability are colored white.

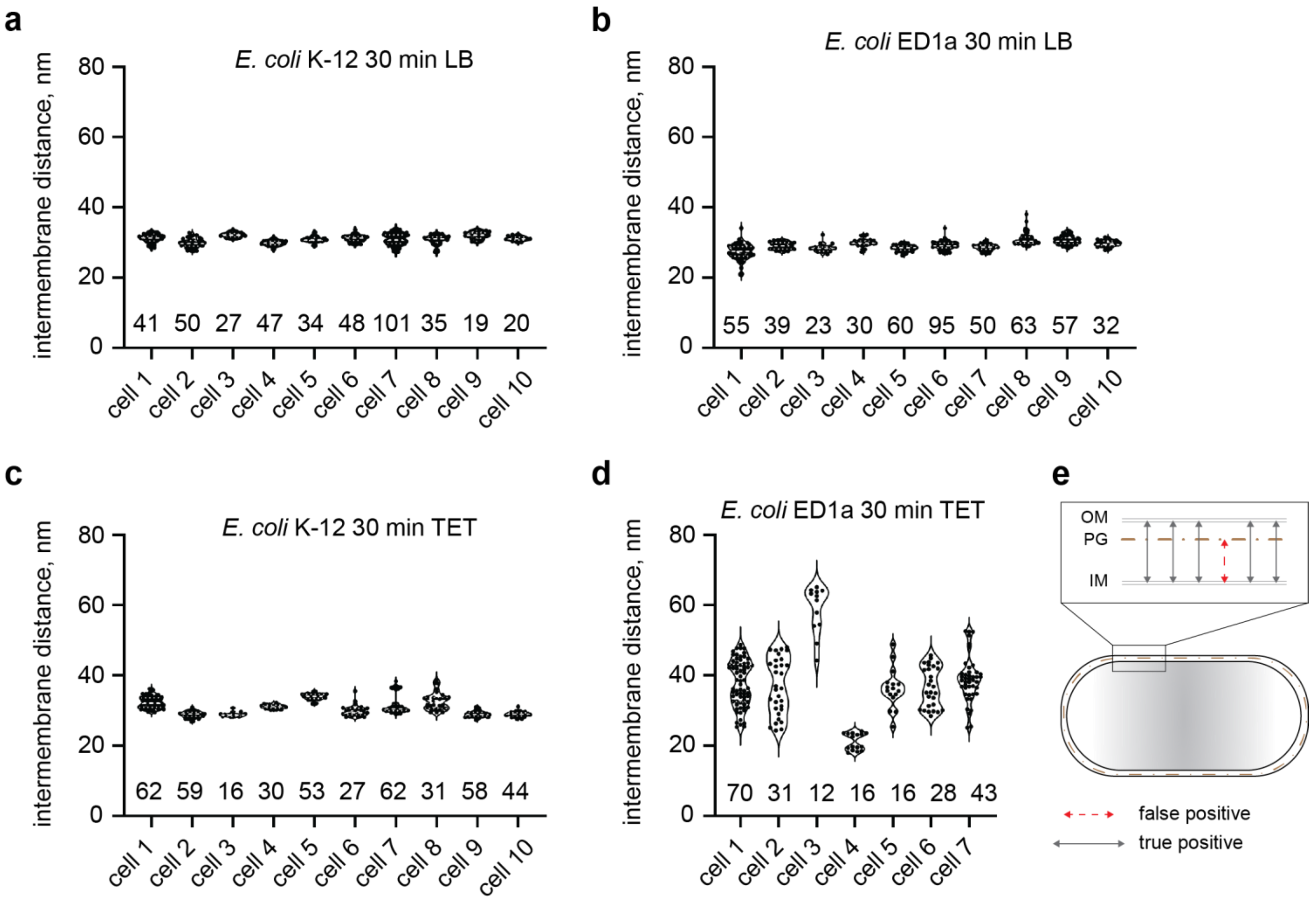

Supplementary Figure 4. Intermembrane distance measurements of cells used for subtomogram averaging indicate membrane wobbling in each individual cell of the ED1a-TET sample.

a – d Measurements of intermembrane distances in individual cells from Fig. 6b. For calculation details, refer to Fig. 6b and the main text. Overall, the thickness of periplasmic space for samples K-12 LB (**a**), ED1a LB (**b**), and K-12 TET (**c**) was constant within each cell and varied slightly between the cells. For *E. coli* ED1 TET sample (**d**), the periplasmic space varied both within single cells and among cells.

e Schematic representation of intermembrane distance measurement. OM – outer membrane, IM – inner membrane, PG – peptidoglycal layer.

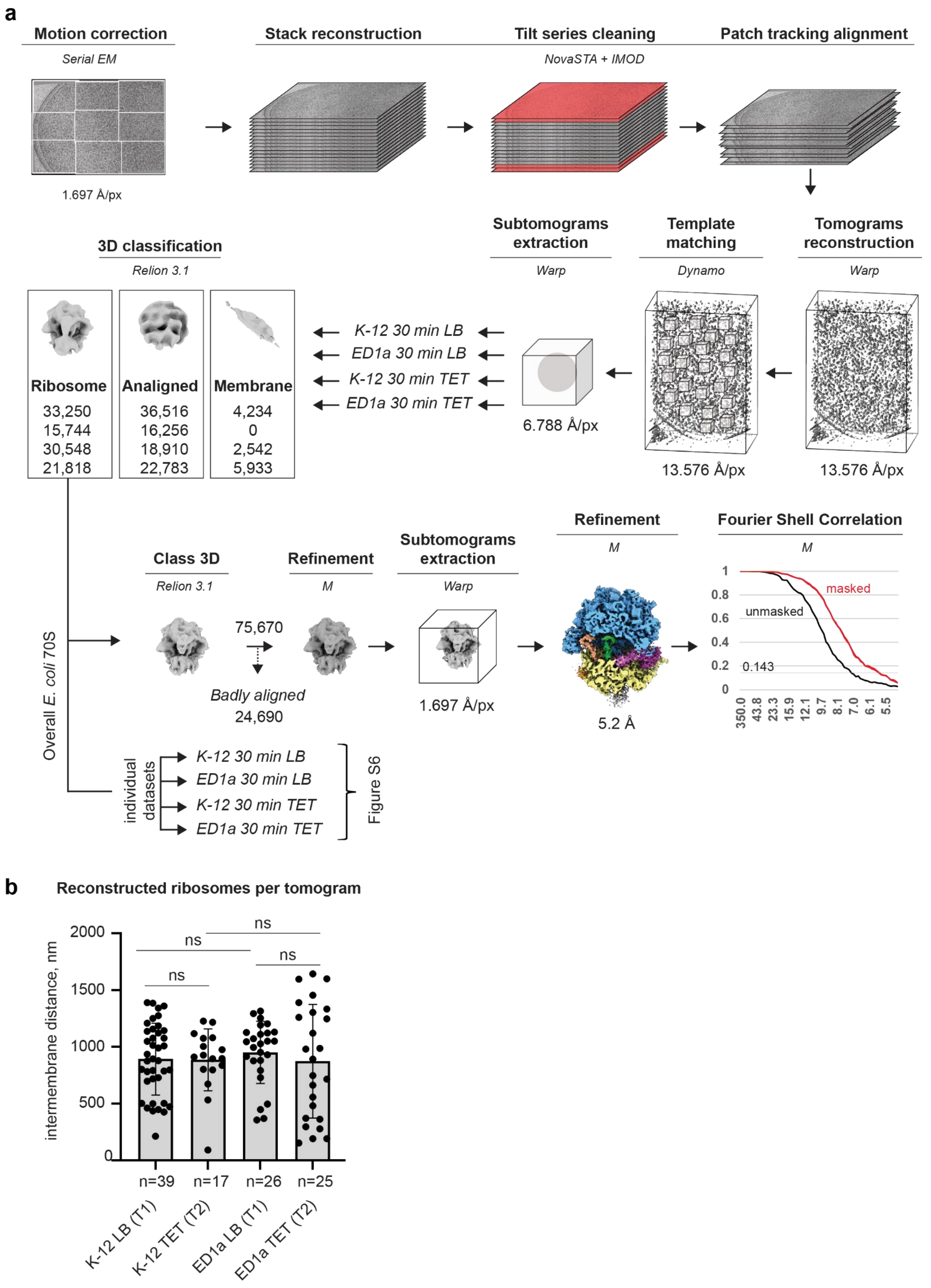
a
Motion correction
Serial EM
1.697 Å/px
Stack reconstruction
Tilt series cleaning
NovaSTA + IMOD
Patch tracking alignment
Tomograms reconstruction
Warp
13.576 Å/px
Template matching
Dynamo
13.576 Å/px
Subtomograms extraction
Warp
6.788 Å/px
K-12 30 min LB
ED1a 30 min LB
K-12 30 min TET
ED1a 30 min TET
3D classification
Relion 3.1
Ribosome
33,250
15,744
30,548
21,818
Analigned
36,516
16,256
18,910
22,783
Membrane
4,234
0
2,542
5,933
Overall E. coli 70S
Class 3D
Relion 3.1
75,670
Badly aligned
24,690
Refinement
M
Subtomograms extraction
Warp
1.697 Å/px
Refinement
M
5.2 Å
Fourier Shell Correlation
M
masked
unmasked
0.143
1
0.8
0.6
0.4
0.2
0
350.0
43.8
23.3
15.9
12.1
9.7
8.1
7.0
6.1
5.5
individual datasets
K-12 30 min LB
ED1a 30 min LB
K-12 30 min TET
ED1a 30 min TET
Figure S6
b
Reconstructed ribosomes per tomogram
intermembrane distance, nm
2000
1500
1000
500
0
ns
ns
ns
ns
n=39
n=17
n=26
n=25
K-12 LB (T1)
K-12 TET (T2)
ED1a LB (T1)
ED1a TET (T2)

Supplementary Figure 5. Applied cryo-ET image processing pipeline achieves high-resolution 70S ribosome structure.

a Image processing of cryo-ET data. Each step is highlighted in bold letters, and software used is denoted in italics. The number of particles is indicated below each density snapshot. The pixel size values reflect data binning and un-binning. All ribosomal subtomograms were merged and aligned in Relion 3.1 and refined in M to an overall structure of 5.2 Å resolution. The detailed scheme of 3D classification used to sort out the structures of 70S with different or ligand occupancies in individual datasets is shown in Supplementary Fig. 2.
b The number of ribosomes per tomogram in each cryo-ET dataset. Only those particles used in the final reconstruction by subtomogram averaging are considered. Each dot corresponds to one tomogram. The statistical significance was calculated using a two-sided unpaired *t*-test.

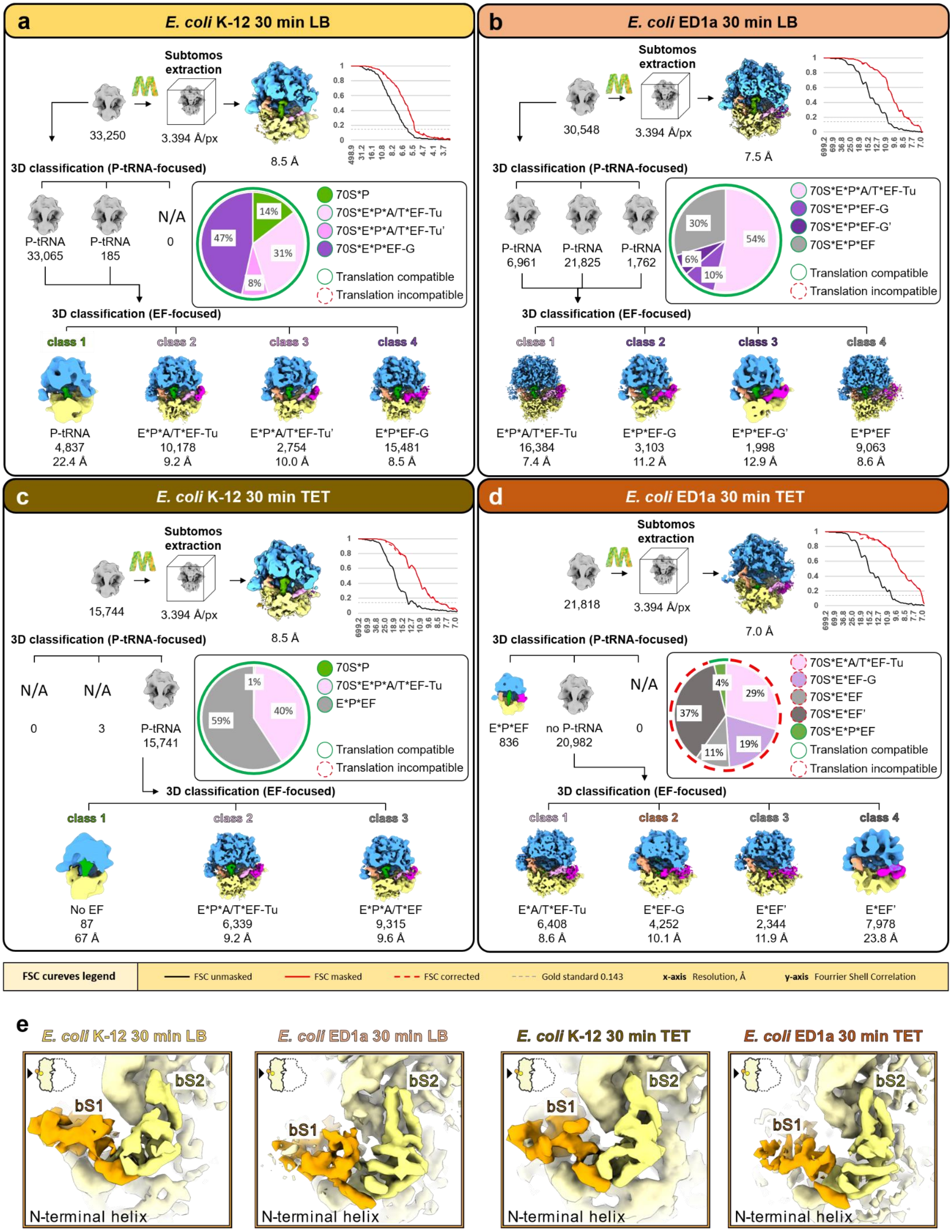
a
E. coli K-12 30 min LB
Subtomos extraction
33,250
3.394 Å/px
8.5 Å
3D classification (P-tRNA-focused)
P-tRNA
33,065
P-tRNA
185
N/A
0
14%
47%
31%
8%
70S*P
70S*E*P*A/T*EF-Tu
70S*E*P*A/T*EF-Tu'
70S*E*P*EF-G
Translation compatible
Translation incompatible
3D classification (EF-focused)
class 1
class 2
class 3
class 4
P-tRNA
4,837
22.4 Å
E*P*A/T*EF-Tu
10,178
9.2 Å
E*P*A/T*EF-Tu'
2,754
10.0 Å
E*P*EF-G
15,481
8.5 Å
b
E. coli ED1a 30 min LB
Subtomos extraction
30,548
3.394 Å/px
7.5 Å
3D classification (P-tRNA-focused)
P-tRNA
6,961
P-tRNA
21,825
P-tRNA
1,762
30%
54%
6%
10%
70S*E*P*A/T*EF-Tu
70S*E*P*EF-G
70S*E*P*EF-G'
70S*E*P*EF
Translation compatible
Translation incompatible
3D classification (EF-focused)
class 1
class 2
class 3
class 4
E*P*A/T*EF-Tu
16,384
7.4 Å
E*P*EF-G
3,103
11.2 Å
E*P*EF-G'
1,998
12.9 Å
E*P*EF
9,063
8.6 Å
c
E. coli K-12 30 min TET
Subtomos extraction
15,744
3.394 Å/px
8.5 Å
3D classification (P-tRNA-focused)
N/A
0
N/A
3
P-tRNA
15,741
1%
40%
59%
70S*P
70S*E*P*A/T*EF-Tu
E*P*EF
Translation compatible
Translation incompatible
3D classification (EF-focused)
class 1
class 2
class 3
No EF
87
67 Å
E*P*A/T*EF-Tu
6,339
9.2 Å
E*P*A/T*EF
9,315
9.6 Å
d
E. coli ED1a 30 min TET
Subtomos extraction
21,818
3.394 Å/px
7.0 Å
3D classification (P-tRNA-focused)
E*P*EF
836
no P-tRNA
20,982
N/A
0
4%
29%
37%
11%
19%
70S*E*A/T*EF-Tu
70S*E*EF-G
70S*E*EF
70S*E*EF'
70S*E*P*EF
Translation compatible
Translation incompatible
3D classification (EF-focused)
class 1
class 2
class 3
class 4
E*A/T*EF-Tu
6,408
8.6 Å
E*EF-G
4,252
10.1 Å
E*EF'
2,344
11.9 Å
E*EF'
7,978
23.8 Å
FSC cureves legend
FSC unmasked
FSC masked
FSC corrected
Gold standard 0.143
x-axis Resolution, Å
y-axis Fourrier Shell Correlation
e
E. coli K-12 30 min LB
E. coli ED1a 30 min LB
E. coli K-12 30 min TET
E. coli ED1a 30 min TET
bS1
bS2
N-terminal helix

Supplementary Figure 6. Subtomogram averaging and 3D classification reveals that the majority of ribosomes are present in a translation-incompetent state in *E. coli* ED1a cells after 30 min of TET treatment.

(A) – (D) Multiple rounds of 3D classification allow identification of 70S structures with different ligand occupancies. The distribution of classes in every sample is shown as a pie chart. The classes containing or lacking tRNA in the P-site are highlighted around the pie charts as green and red circles, respectively. The classes with ambiguous density at the elongation factor binding site are captioned as EF.

(E) Segmented density of the r-protein bS1 (orange) anchored with its N-terminal helix to the r-protein bS2 (yellow) on the small ribosomal subunits of 70S averages from *E .coli* K-12 30 min LB, *E. coli* K-12 30 min TET, *E. coli* ED1a 30 min LB, *E. coli* ED1a 30 min TET.

Supplementary References

1 Paternoga, H. *et al.* Structural conservation of antibiotic interaction with ribosomes. *Nat Struct Mol Biol* **30**, 1380-1392, doi:10.1038/s41594-023-01047-y (2023).

2 Cocozaki, A. I. *et al.* Resistance mutations generate divergent antibiotic susceptibility profiles against translation inhibitors. *Proc Natl Acad Sci U S A* **113**, 8188-8193, doi:10.1073/pnas.1605127113 (2016).

3 Jenner, L. *et al.* Structural basis for potent inhibitory activity of the antibiotic tigecycline during protein synthesis. *Proc Natl Acad Sci U S A* **110**, 3812-3816, doi:10.1073/pnas.1216691110 (2013).
