# Supplementary material for: Bactericidal effect of tetracycline in E. coli strain ED1a may be associated with ribosome dysfunction
Source: Nat Commun. 2024 Jun 5;15:4783. doi: 10.1038/s41467-024-49084-5 (PMC11153495; doi:10.1038/s41467-024-49084-5)
Supplement: Supplementary file 3 — Description of Additional Supplementary Files [file 41467_2024_49084_MOESM3_ESM.pdf]

File name: Supplementary Data 1
Description: Thermal proteome profiling hits. A list of the proteins identified by thermal proteome profiling with peptide coverage, gene ontology annotations, abundance and stability scores, their respective effect size, and p-values, for timepoints T0, T1, T2, T3, and T4, for both strains.
