# Supplementary material for: Bactericidal effect of tetracycline in E. coli strain ED1a may be associated with ribosome dysfunction
Source: Nat Commun. 2024 Jun 5;15:4783. doi: 10.1038/s41467-024-49084-5 (PMC11153495; doi:10.1038/s41467-024-49084-5)
Supplement: Supplementary file 5 — Reporting Summary [file 41467_2024_49084_MOESM5_ESM.pdf]

nature portfolio

Corresponding author(s): Martin Beck

Last updated by author(s): May 23, 2024

# Reporting Summary

Nature Portfolio wishes to improve the reproducibility of the work that we publish. This form provides structure for consistency and transparency in reporting. For further information on Nature Portfolio policies, see our Editorial Policies and the Editorial Policy Checklist.

## Statistics

For all statistical analyses, confirm that the following items are present in the figure legend, table legend, main text, or Methods section.

| n/a | Confirmed | |
|---|---|---|
| ☐ | ☒ | The exact sample size (*n*) for each experimental group/condition, given as a discrete number and unit of measurement |
| ☐ | ☒ | A statement on whether measurements were taken from distinct samples or whether the same sample was measured repeatedly |
| ☐ | ☒ | The statistical test(s) used AND whether they are one- or two-sided<br>*Only common tests should be described solely by name; describe more complex techniques in the Methods section.* |
| ☒ | ☐ | A description of all covariates tested |
| ☐ | ☒ | A description of any assumptions or corrections, such as tests of normality and adjustment for multiple comparisons |
| ☐ | ☒ | A full description of the statistical parameters including central tendency (e.g. means) or other basic estimates (e.g. regression coefficient) AND variation (e.g. standard deviation) or associated estimates of uncertainty (e.g. confidence intervals) |
| ☐ | ☒ | For null hypothesis testing, the test statistic (e.g. $F$, $t$, $r$) with confidence intervals, effect sizes, degrees of freedom and $P$ value noted<br>*Give P values as exact values whenever suitable.* |
| ☒ | ☐ | For Bayesian analysis, information on the choice of priors and Markov chain Monte Carlo settings |
| ☒ | ☐ | For hierarchical and complex designs, identification of the appropriate level for tests and full reporting of outcomes |
| ☒ | ☐ | Estimates of effect sizes (e.g. Cohen's *d*, Pearson's *r*), indicating how they were calculated |

*Our web collection on statistics for biologists contains articles on many of the points above.*

## Software and code

Policy information about availability of computer code

| | |
|---|---|
| Data collection | Electron tomography: SerialEM version 3.81; Single-particle cryo-EM: EPU v2.14 (Thermo Scientific); Proteomics: Proteome discoverer v2.4, Mass Spectrometer Software XCalibur.<br>Details of data collection parameters are described in Methods sections of the manuscript. |
| Data analysis | IMOD (v 4.10.9 and 4.11.5); Warp / M v 1.0.9; dynamo2m toolbox (https://github.com/alisterburt/dynamo2m); NovaSTA package (Turoňová, Zenodo, 2022 https://github.com/turonova/novaSTA); Dynamo; Relion 3.1.3; CryoSPARC v3.3.1; Coot v0.8.9.2; UCSF ChimeraX v1.4; Fiji v1.53; Proteome discoverer 4.2; R v3.6.1 (2019-07-05), Python 3.6.10 \|Anaconda, Inc.\| (default, Mar 25 2020); GraphPad Prism 9.0; Microsoft Excel 2019<br>Details on data analysis software are described in Methods sections of the manuscript. |

For manuscripts utilizing custom algorithms or software that are central to the research but not yet described in published literature, software must be made available to editors and reviewers. We strongly encourage code deposition in a community repository (e.g. GitHub). See the Nature Portfolio guidelines for submitting code & software for further information.

# Data

Policy information about availability of data

All manuscripts must include a data availability statement. This statement should provide the following information, where applicable:

- Accession codes, unique identifiers, or web links for publicly available datasets
- A description of any restrictions on data availability
- For clinical datasets or third party data, please ensure that the statement adheres to our policy

Cryo-ET density maps generated in this study have been deposited in the EM Data Bank with the following accession codes: EMD-18036 [https://www.ebi.ac.uk/emdb/EMD-18036] (in situ E. coli 70S ribosome), EMD-18037 [https://www.ebi.ac.uk/emdb/EMD-18037] (in situ 70S ribosome of E. coli K-12 untreated cells), EMD-18038 [https://www.ebi.ac.uk/emdb/EMD-18038] (in situ 70S ribosome of E. coli K-12 cells treated with tetracycline), EMD-18039 [https://www.ebi.ac.uk/emdb/EMD-18039] (in situ 70S ribosome of E. coli ED1a untreated cells), EMD-18040 [https://www.ebi.ac.uk/emdb/EMD-18040] (in situ 70S ribosome of E. coli ED1a cells treated with tetracycline), EMD-18041 [https://www.ebi.ac.uk/emdb/EMD-18041] (E. coli K-12 70S ribosome bound to mRNA A-tRNA, P-tRNA, E-tRNA), EMD-18042 [https://www.ebi.ac.uk/emdb/EMD-18042] (E. coli ED1a 70S ribosome bound to mRNA A-tRNA, P-tRNA, E-tRNA), EMDB-19206 [https://www.ebi.ac.uk/emdb/EMD-19206] (E. coli ED1a 70S·TET 30S head focused), EMDB-19207 [https://www.ebi.ac.uk/emdb/EMD-19207] (E. coli ED1a 70S·TET 30S body focused), EMDB-19208 [https://www.ebi.ac.uk/emdb/EMD-19208] (E. coli ED1a 70S·TET 50S focused). Cryo-ET raw tilt series have been deposited in the EM Public Image Archive (EMPIAR) with the following accession codes: EMPIAR-11945 (E. coli K-12 untreated cells), EMPIAR-11946 (E. coli K-12 tetracycline-treated cells), EMPIAR-11947 (E. coli ED1a untreated cells), EMPIAR-11948 (E. coli ED1a tetracycline-treated cells). The ribosome models that were used for interpretation of obtained maps and structural comparisons were downloaded from the protein data bank (PDB) under following accession numbers: 4V9A, 5J5B, 7K00, 8CF1. All proteomics data associated with this manuscript have been uploaded to the PRIDE online repository (https://www.ebi.ac.uk/pride/) with the identifier PXD044697. Source data are provided with this paper.

# Research involving human participants, their data, or biological material

Policy information about studies with human participants or human data. See also policy information about sex, gender (identity/presentation), and sexual orientation and race, ethnicity and racism.

| | |
|---|---|
| Reporting on sex and gender | Study does not involve human research participants |
| Reporting on race, ethnicity, or other socially relevant groupings | Study does not involve human research participants |
| Population characteristics | Study does not involve human research participants |
| Recruitment | Study does not involve human research participants |
| Ethics oversight | Study does not involve human research participants |

Note that full information on the approval of the study protocol must also be provided in the manuscript.

# Field-specific reporting

Please select the one below that is the best fit for your research. If you are not sure, read the appropriate sections before making your selection.

☒ Life sciences ☐ Behavioural & social sciences ☐ Ecological, evolutionary & environmental sciences

For a reference copy of the document with all sections, see nature.com/documents/nr-reporting-summary-flat.pdf

# Life sciences study design

All studies must disclose on these points even when the disclosure is negative.

| | |
|---|---|
| Sample size | No prior assumptions were made regarding sample size. The maximum amount of cryo-EM and cryo-ET data were acquired within the available microscope time. |
| Data exclusions | For cryo-ET analysis, a fraction of data was excluded due to acquisition problems or poor signal-to-noise ratio of collected tilt series which led to problems with alignment and tomogram reconstruction of such data. |
| Replication | All microbiology experiments analyses were performed at least in three biological replicates. MS analysis was performed using four biological replicates. Cryo-ET studies were performed on cells prepared from at least three cultures grown in different days. All replicates were successful. |
| Randomization | was not needed for the type of experiments performed in this study |
| Blinding | was not needed for the type of experiments performed in this study |

# Reporting for specific materials, systems and methods

We require information from authors about some types of materials, experimental systems and methods used in many studies. Here, indicate whether each material, system or method listed is relevant to your study. If you are not sure if a list item applies to your research, read the appropriate section before selecting a response.

## Materials & experimental systems

| n/a | Involved in the study | |
|---|---|---|
| ☒ | ☐ | Antibodies |
| ☒ | ☐ | Eukaryotic cell lines |
| ☒ | ☐ | Palaeontology and archaeology |
| ☐ | ☒ | Animals and other organisms |
| ☒ | ☐ | Clinical data |
| ☒ | ☐ | Dual use research of concern |
| ☒ | ☐ | Plants |

## Methods

| n/a | Involved in the study | |
|---|---|---|
| ☒ | ☐ | ChIP-seq |
| ☒ | ☐ | Flow cytometry |
| ☒ | ☐ | MRI-based neuroimaging |

## Animals and other research organisms

Policy information about studies involving animals; ARRIVE guidelines recommended for reporting animal research, and Sex and Gender in Research

| | |
|---|---|
| Laboratory animals | The study did not involve laboratory animals |
| Wild animals | The study did not involve wild animals |
| Reporting on sex | Sex was not considered in the study design, as the work was performed only using Escherichia coli cells |
| Field-collected samples | The study did not involve field-collected samples |
| Ethics oversight | No ethical approval or guidance was required, as the work was performed only using Escherichia coli cells |

Note that full information on the approval of the study protocol must also be provided in the manuscript.
